# Internet Survey Evaluation of Demographic Risk Factors for Injury in Canine Agility Athletes

**DOI:** 10.3389/fvets.2022.869702

**Published:** 2022-04-08

**Authors:** Annika E. Sundby, Arielle Pechette Markley, Abigail B. Shoben, Nina R. Kieves

**Affiliations:** ^1^Department of Veterinary Clinical Sciences, College of Veterinary Medicine, Ohio State University, Columbus, OH, United States; ^2^Veterinary Medical Center, College of Veterinary Medicine, Ohio State University, Columbus, OH, United States; ^3^Division of Biostatistics, College of Public Health, The Ohio State University, Columbus, OH, United States

**Keywords:** agility, dog, canine, sports medicine, injury, demographics

## Abstract

**Objective:**

The purpose of this study was to compare previously identified demographic risk factors for injury in agility dogs, and explore other potential associations with demographic risk factors in new populations, and across different levels of injury severity.

**Procedures:**

An internet-based survey of agility handlers was conducted. The primary outcome was if the dog had ever had an injury that kept from agility for over a week. Demographic information about the dog and handler were recorded. Logistic regression was used to quantify associations between variables of interest with injury history and all models were adjusted for age. Analyses were stratified by geographic location. Final model building was done *via* backward selection.

**Results:**

The sample included 2,962 dogs from North America and 1,235 dogs from elsewhere. In the North American sample, 8 variables were associated with injury history; dog breed, height and weight, handler age, gender, agility experience, competing at the national level, age dog was acquired, and taking radiographs to assess growth plate closure. In the non-North American sample, 4 variables were associated with injury history; breed, handler age, occupation (dog trainer or not), and handler medical training. In both samples, Border Collies showed a marked increase in injury risk (ORs 1.89 and 2.34) and handler age >65 was associated with lower risk (ORs 0.62 and 0.77). Consistent with previous studies, greater handler experience was associated with reduced risk in the North American sample, but the other sample did not show this pattern, even in unadjusted models. Dog spay/neuter status was not associated with injury risk in either sample.

**Conclusions and Clinical Relevance:**

Dogs with radiographs assessing growth plate closure may have increased injury risk as this population of owners may plan to train their dog harder, and at an earlier age. This finding also poses the question of whether or not growth plate closure is a good indicator of safety for increasing training intensity. Knowledge of what risk factors exist for injury in agility dogs is imperative in determining direction for future prospective studies, as well as creating recommendations to help prevent injury in this population of dogs.

## Introduction

Canine agility is a sport where a handler directs their dog through a course of pre-set obstacles, such as jumps, weave poles, tunnels, A-frames, teeters, etc., which need be completed within a specific time limit. Entries into events sponsored by the American Kennel Club have increased 38% (870,603 to 1,202,711) from 2009 and 2019, indicating a dramatic increase in the sport's popularity.^1^ There has been an increase in reported injury rates in agility dogs, as demonstrated in a recent study from 2019 that reported an overall injury rate of 41.7%, a substantial increase from the 32% rate reported in 2009 (1, 2). The increase in popularity, competitiveness and injury rates indicates a need for updated and expanded information regarding risk factors for injury to these canine athletes.

Despite the increase in popularity and numerous changes that have occurred in canine agility in the past decade, there has been little updated information in regards to risk factors for injuries sustained by agility dogs. A previous study evaluating demographic risk factors for injury in agility dogs, published in 2013, by Cullen et al., reported that the Border Collie breed and <4 years of agility experience for dogs were associated with an increased risk of injury (1). The study also found that dogs having >4 years agility experience, and handlers with 5–10 years or >10 years handling experience were associated with a decreased risk of injury (1). A more recent study by Evanow et al. found that Border Collie breed, increased age, early spay/neuter and higher level of competition were associated with increased injury risk (3).

Both the Cullen et al. and Evanow et al. studies were done nearly exclusively in North American (United States and Canada) samples, raising the issue of whether or not risk factors for injuries might be different for dogs competing in agility in different geographic regions. Differences in injury type and injury frequency among agility dogs in different geographic regions have been previously reported (2), making it likely that there are also differences in risk factors between geographic regions. In greyhound racing, demographic risk factors for injury have been reported, and these demographic risk factors for injury have also been shown to vary by country and racing jurisdiction (4). Geographical comparisons of injury risks have not been evaluated in any other canine athlete population, including agility.

The purpose of this study was to investigate risk factors for injury in dogs competing in agility, focusing on handler and dog demographics, comparing previously identified risk factors and exploring potential associations with demographic factors in new populations and with different levels of injury severity. We hypothesized that less experienced handlers, competing at a higher level, and early spay/neuter would increase the risk of developing an injury. Based on differences observed in injury type and frequency by geographic region (2), we also hypothesized that demographic risk factors would vary by geographic region.

## Materials and Methods

An internet-based survey (Qualtrics survey software, Provo UT) was conducted during a 6-week period in the fall of 2019. The details of this survey have been previously published (2). Briefly, participants who had at least one dog competing in agility in the past 3 years were eligible to fill out the survey. If participants had more than one dog that was eligible for inclusion, alphabetical order was used to select the dog for which the survey was completed (i.e., dog with name closest to letter “A”). Completion of the survey for multiple dogs was permitted, although this analysis was performed using only the first dog for each participant. The Ohio State University Institutional Review Board reviewed and approved the research protocol and survey.

Survey questions asked about demographic information for both the dog and the handler. Demographic information about the dog included age, height, weight, breed, sex/neuter status, country, age when acquired, from where the dog was acquired, if it was acquired with agility in mind, and if agility was the main sport focus. Handler demographics recorded included age, gender, education, profession (dog training professionally vs. not), medical training (veterinary, human, or none), and handler agility experience.

The primary outcome was history of any injury, defined as an injury that kept the dog from participating in agility for over a week, as reported by the owner. A secondary outcome, history of “severe injury” was defined as any injury where the dog was out of agility for more than 3 months.

Descriptive statistics (means and proportions) were first calculated for all variables. Separate logistic regression models were used to quantify associations between variables with the binary injury status outcome variables. All models were age adjusted to account for the differences in lifetime exposure for different aged dogs. For categorical variables, a multivariate Wald test was used to test for the overall association between that variable and injury history. In some cases, categories of responses were collapsed in order to avoid small cell sizes and facilitate interpretation; such adjustments were made prior to final model building and without respect to the association between the variable and the outcome of interest.

Risk factors were identified through backward stepwise selection in all models. The primary model used all available data and the outcome of any injury history for comparison with previous studies. To evaluate potential differences in risk factors between North American and non-North American agility dogs, models for risk of any injury were also constructed separately in the North American and non-North American subsamples. A final model evaluated risk factors for severe injury using all available data but with the outcome of severe injury (out of agility for longer than 3 months).

For all models, variables that were significant at p < 0.20 in models adjusted only for age of the dog were retained for variable selection. All variables retained from the initial selection process were included in the first adjusted model and then stepwise backward selection was used to eliminate the variable with the highest *p*-value until all variables in the model had p < 0.05. Data were analyzed using Stata 15.1 (College Station, TX).

## Results

The sample included data from 4,197 dogs, including 2,962 from North American and 1,235 from the rest of the world (Table 1). Injury keeping the dog from participating in agility for a week or longer was reported in 1,739 (41.4%) dogs total, with a higher rate reported in the non-North American sample (560 injured, 45.3%) compared to the North American sample (1,179 injured, 39.8%). Associations of all variables after adjusting for dog age are provided in Supplementary Tables 1, 2.

**Table 1:** Descriptive statistics of the sample (*n* = 4,197 dogs and handlers).

	**Full sample**	**North American**	**Non-NA**
	**(*n* = 4,197)**	**sample**	**sample**
		**(*n* = 2,962)**	**(*n* = 1,235)**
**Dog demographics**			
Dog age (years)^*^	6.3 (2.9)	6.5 (3.0)	5.8 (2.7)
Dog height (inches)^*^	18.2 (4.3)	18.3 (4.4)	18.0 (4.1)
Dog weight (pounds)^*^	36.6 (17.6)	37.2 (18.4)	35.1 (15.6)
**Breed**			
Border Collie	934 (22.3)	565 (19.1)	369 (29.9)
Mixed breed	555 (13.2)	380 (12.8)	175 (14.2)
Shetland Sheepdog	277 (6.6)	203 (6.9)	74 (6.0)
Australian Shephard	285 (6.8)	244 (8.2)	41 (3.3)
Other	2,146 (51.1)	1,570 (53.0)	576 (46.6)
**Country/Region**			
United States	2,570 (61.2)	2,570 (86.8)	0 (0.0)
Canada	392 (9.3)	392 (13.2)	0 (0.0)
UK/Ireland	469 (11.2)	0 (0.0)	469 (38.0)
Cont. Europe	343 (8.2)	0 (0.0)	343 (27.8)
Australia	163 (3.9)	0 (0.0)	163 (13.2)
Other	260 (6.2)	0 (0.0)	260 (21.1)
**Age brought dog home**			
<8 weeks	868 (20.7)	602 (20.4)	266 (21.6)
8–12 weeks	2,246 (53.6)	1,545 (52.3)	701 (56.9)
13–15 weeks	230 (5.5)	164 (5.6)	66 (5.4)
4–6 months	241 (5.8)	171 (5.8)	70 (5.7)
7–12 months	238 (5.7)	186 (6.3)	52 (4.2)
>12 months	366 (8.7)	288 (9.7)	78 (6.3)
**How acquired**			
Breeder	3,092 (73.8)	2,170 (73.4)	922 (74.7)
Rescue/Shelter	708 (16.9)	527 (17.8)	181 (14.7)
Other	389 (9.3)	258 (8.7)	131 (10.6)
**Acquired w/agility in mind**			
No	1,214 (29.0)	787 (26.6)	427 (34.6)
Yes	2,978 (71.0)	2,171 (73.4)	807 (65.4)
**Agility main sport focus**			
Yes	3,016 (71.9)	2,075 (70.1)	941 (76.2)
Mostly	824 (19.6)	611 (20.6)	213 (17.3)
No	356 (8.5)	275 (9.3)	81 (6.6)
**Sex/neuter status**			
Male, intact	671 (16.9)	439 (15.7)	232 (19.5)
Female, intact	486 (12.2)	295 (10.6)	191 (16.1)
Male, neutered <10 months	377 (9.5)	285 (10.2)	92 (7.7)
Male, neutered 10–18 months	538 (13.5)	396 (14.2)	142 (12.0)
Male, neutered >24 months	421 (10.6)	312 (11.2)	109 (9.2)
Female, spayed <1 cycle	539 (13.6)	411 (14.7)	128 (10.8)
Female, spayed 1 cycle	367 (9.2)	235 (8.4)	132 (11.1)
Female, spayed >1 cycle	580 (14.6)	418 (15.0)	162 (13.6)
**Front dew claws**			
Intact	2,951 (70.4)	1,833 (62.0)	1,118 (90.6)
Removed	1,170 (27.9)	1,078 (36.5)	92 (7.5)
Unknown	70 (1.7)	46 (1.6)	24 (1.9)
**Rear dew claws**			
Intact	782 (18.7)	378 (12.8)	404 (32.8)
Removed or born without	3,240 (77.4)	2,466 (83.4)	774 (62.9)
Unknown	165 (3.9)	112 (3.8)	53 (4.3)
**Docked tail**			
Yes	760 (18.1)	698 (23.6)	62 (5.0)
No/unknown	3,435 (81.9)	2,263 (76.4)	1,172 (95.0)
**Growth plate x-rays**			
Not done	3,432 (81.8)	2,336 (78.9)	1,096 (88.8)
Done at least once	763 (18.2)	625 (21.1)	138 (11.2)
**Handler demographics**			
**Handler current age**			
18–24	208 (5.0)	92 (3.1)	116 (9.5)
25–34	657 (15.7)	358 (12.1)	299 (24.4)
35–44	634 (15.2)	370 (12.5)	264 (21.6)
45–54	866 (20.8)	608 (20.6)	258 (21.1)
55–64	1,176 (28.2)	974 (33.0)	202 (16.5)
65+	633 (15.2)	548 (18.6)	85 (6.9)
**Handler gender**			
Female	3,915 (93.8)	2,782 (94.4)	1,133 (92.5)
Male	212 (5.1)	131 (4.4)	81 (6.6)
Other gender identity	46 (1.1)	35 (1.2)	11 (0.9)
**Handler education**			
Graduate or professional degree	1,389 (33.5)	995 (33.9)	394 (32.5)
4-year college	1,296 (31.2)	1,005 (34.2)	291 (24.0)
2-year college	452 (10.9)	352 (12.0)	100 (8.3)
Some college	586 (14.1)	408 (13.9)	178 (14.7)
HS degree (or less)	425 (10.3)	177 (6.0)	248 (20.5)
**Handler profession**			
Not a dog trainer	2,738 (66.1)	650 (22.2)	404 (33.1)
Paid trainer, not primary job	1,054 (25.4)	277 (9.5)	75 (6.2)
Professional trainer	352 (8.5)	1,997 (68.3)	741 (60.7)
**Handler medical training/experience**			
None of these	3,215 (77.9)	2,230 (76.4)	985 (81.4)
Veterinarian	149 (3.6)	105 (3.6)	44 (3.6)
Licensed vet tech	106 (2.6)	90 (3.1)	16 (1.3)
Veterinary assistant	96 (2.3)	66 (2.3)	30 (2.5)
Human health care professional	562 (13.6)	427 (14.6)	135 (11.2)
**Handler agility experience**			
<3 years	410 (9.8)	231 (7.8)	179 (14.5)
3–5 years	722 (17.2)	457 (15.5)	265 (21.5)
6–10 years	1,054 (25.2)	737 (24.9)	317 (25.7)
11–15 years	696 (16.6)	514 (17.4)	182 (14.8)
>15 years	1,308 (31.2)	1,018 (34.4)	290 (23.5)
**Handler competed at national level**			
No	1,893 (45.2)	1,349 (45.6)	544 (44.1)
Yes	2,299 (54.8)	1,610 (54.4)	689 (55.9)
**Handler competed at international level**		
No	3,744 (89.5)	2,761 (93.5)	983 (79.9)
Yes	439 (10.5)	191 (6.5)	248 (20.2)

(^*^)*Values are N (%) except for continuous variables which are means (SD)*.

Among the entire sample, the final model for injury history included nine predictor variables in addition to dog age (Table 2). As previously reported, Border Collies were much more likely to report injury history than all other breeds, and as in previous studies, dogs of handlers with the most experience (>15 years) were the least likely to have an injury history, although the differences among other categories of agility experience were small.

**Table 2:** Coefficients from final adjusted model of risk factors of any injury using the full sample.

	**Adjusted OR (95% CI)**	**Adjusted *p*-value**
Dog age (per 1 year older)	1.17 (1.14, 1.20)	<0.001
**Height & weight together**		<0.001
Dog height (per 4 inches taller)	0.83 (0.73, 0.95)	
Dog weight (per 10 pounds heavier)	1.17 (1.08, 1.27)	
**Breed**		<0.001
Border collie	1.97 (1.64, 2.37)	
Mixed breed	1.03 (0.83, 1.28)	
Shetland sheepdog	1.18 (0.89, 1.56)	
Australian shephard	0.98 (0.75, 1.29)	
Other	Reference	
**Age brought dog home**		0.014
<8 weeks	0.99 (0.83, 1.17)	
8–12 weeks	Reference	
13–15 weeks	1.01 (0.75, 1.37)	
4–6 months	0.98 (0.73, 1.32)	
7–12 months	0.97 (0.72, 1.30)	
>12 months	0.62 (0.48, 0.80)	
**Agility main sport focus**		0.048
Yes	Reference	
Mostly	1.07 (0.90, 1.27)	
No	0.76 (0.59, 0.98)	
**Growth plate x-rays**		0.025
Not done	Reference	
Done at least once	1.22 (1.03, 1.46)	
**Handler current age**		<0.001
18–24	Reference	
25–34	1.30 (0.92, 1.86)	
35–44	1.27 (0.89, 1.82)	
45–54	1.08 (0.76, 1.53)	
55–64	0.92 (0.65, 1.30)	
65+	0.59 (0.41, 0.86)	
**Handler medical training/experience**		0.005
None of these	Reference	
Veterinarian	0.53 (0.36, 0.77)	
Licensed vet tech	0.68 (0.44, 1.05)	
Veterinary assistant	1.19 (0.77, 1.82)	
Human health care professional	0.90 (0.74, 1.09)	
**Handler agility experience**		0.035
<3 years	1.12 (0.84, 1.51)	
3–5 years	1.20 (0.96, 1.50)	
6–10 years	1.32 (1.09, 1.59)	
11–15 years	1.27 (1.04, 1.56)	
>15 years	Reference	
**Handler competed at national level**		0.007
No	Reference	
Yes	1.23 (1.06, 1.43)	

Other variables associated with injury history have not been previously reported. Dogs whose handlers were over the age of 65 had the lowest odds of injury history, with the highest odds observed among handlers 25–34 and 35–44 years old. Dogs of handlers who reported having ever competed at the national level in agility had higher risk of injury, while dogs of handlers who are veterinarians or licensed veterinary technicians had lower odds of injury compared to other categories of occupation (Table 2). Dogs that were acquired at >12 months of age were less likely to report an injury history, even controlling for age and other variables in the final model, while there was little difference observed among different age categories below this threshold (Table 2). Dogs for whom agility was not the main sport focus were also less likely to have been injured, while dogs who had radiographs done to assess growth plate closure were at higher risk. Finally, owner-reported dog height and weight were jointly associated with injury history. Dog height was negatively associated with injury history, while dog weight was positively associated with injury history, indicating that among dogs of the same height, the odds of injury were greater for dogs who were heavier, controlling for breed and all other variables in the model.

In the North American sample, most of the same patterns were observed, with the final model including all the same variables except handler medical training and whether agility was the main sport focus of the dog (Table 3). In the North American sample, handler gender was included in the final model, with dogs of male handlers less likely to report injury.

**Table 3:** Coefficients from final adjusted model of risk factors of any injury using the North American sample.

	**Adjusted OR (95% CI)**	**Adjusted *p*-value**
Dog age (per 1 year older)	1.17 (1.14, 1.20)	<0.001
**Height & weight together**		0.002
Dog height (per 4 inches taller)	0.84 (0.72, 0.99)	
Dog weight (per 10 pounds heavier)	1.17 (1.06, 1.29)	
**Breed**		<0.001
Border collie	1.89 (1.51, 2.37)	
Mixed breed	0.85 (0.66, 1.10)	
Shetland sheepdog	1.28 (0.93, 1.77)	
Australian shephard	0.95 (0.70, 1.28)	
Other	Reference	
**Age brought dog home**		0.001
<8 weeks	0.99 (0.81, 1.22)	
8–12 weeks	Reference	
13–15 weeks	1.20 (0.85, 1.70)	
4–6 months	1.01 (0.72, 1.43)	
7–12 months	1.14 (0.82, 1.59)	
>12 months	0.60 (0.45, 0.81)	
**Growth plate x-rays**		0.020
Not done	Reference	
Done at least once	1.26 (1.04, 1.53)	
**Handler current age**		<0.001
18–24	Reference	
25–34	1.17 (0.71, 1.94)	
35–44	1.25 (0.76, 2.07)	
45–54	1.13 (0.69, 1.83)	
55–64	0.93 (0.57, 1.49)	
65+	0.62 (0.38, 1.03)	
**Handler gender**		0.044
Female	Reference	
Male	0.64 (0.43, 0.94)	
Non-binary/differently identify	0.65 (0.31, 1.37)	
**Handler agility experience**		0.035
<3 years	1.12 (0.84, 1.51)	
3–5 years	1.20 (0.96, 1.50)	
6–10 years	1.32 (1.09, 1.59)	
11–15 years	1.27 (1.04, 1.56)	
>15 years	Reference	
**Handler competed at national level**		0.014
No	Reference	
Yes	1.25 (1.05, 1.49)	

In the non-North American sample, only four variables (in addition to dog age) were retained in the final model (Table 4). Border Collies were again more likely to have been injured, with an even higher odds ratio than in the North American sample, and, unlike in North America, Mixed Breed dogs were also at higher risk of injury relative to other non-Border Collie breeds (Table 4). Handler age was also again associated with injury risk, with dogs of older handlers (65+) at lower risk and dogs of handlers 25–44 at higher risk. Dogs of veterinarians and licensed veterinary technicians were at significantly decreased risk of injury history in this sample. Finally, unlike in the North American sample, dogs of handlers who reported that they were a professional dog trainer or that they were a trainer to others (if not their primary job) were at higher risk of injury in the non-North American sample.

**Table 4:** Coefficients from final adjusted model of risk factors of any injury using the non-North American sample.

	**Adjusted OR (95% CI)**	**Adjusted *p*-value**
Dog Age (per 1 year old)	1.20 (1.14, 1.25)	<0.001
**Breed**		<0.001
Border collie	2.34 (1.76, 3.11)	
Mixed breed	1.50 (1.04, 2.15)	
Shetland sheepdog	0.95 (0.56, 1.61)	
Australian shephard	1.26 (0.65, 2.46)	
Other	Reference	
**Handler profession**		0.003
Not a dog trainer	Reference	
Paid trainer, not primary job	1.54 (1.18, 2.00)	
Professional trainer	1.53 (0.91, 2.56)	
**Handler current age**		0.039
18–24	Reference	
25–34	1.52 (0.95, 2.43)	
35–44	1.40 (0.86, 2.26)	
45–54	0.99 (0.61, 1.60)	
55–64	1.05 (0.63, 1.73)	
65+	0.77 (0.41, 1.43)	
**Handler medical training/experience**		0.029
None of these	Reference	
Veterinarian	0.40 (0.21, 0.79)	
Licensed vet tech	0.38 (0.12, 1.19)	
Veterinary assistant	1.04 (0.49, 2.23)	
Human health care professional	0.77 (0.52, 1.13)	

Severe injury keeping the dog out for 4–6 months or longer (>3 months) was reported for 629 dogs, representing 15.0% of all dogs in the survey and 36.2% of the 1,739 dogs reporting any injury history. After model building, four variables (in addition to dog age) remained in the model with severe injury as the outcome: breed, history of radiographs to assess growth plate closure, handler age, and handler medical training (Table 5). As in the model for any injury, Border Collies were at significantly higher risk of severe injury compared to all other breeds. Dogs who had had radiographs done to assess growth plate closure were also at higher risk of severe injury. The oldest handlers (65+) were at lowest risk of reporting a severe injury to their dog and lower risk was also observed among handlers who were also veterinarians.

**Table 5:** Coefficients from final adjusted model of risk factors of severe injury.

	**Adjusted OR (95% CI)**	**Adjusted *p*-value**
Dog Age (per 1 year old)	1.21 (1.17, 1.25)	<0.001
**Breed**		<0.001
Border collie	1.70 (1.37, 2.09)	
Mixed breed	0.84 (0.63, 1.13)	
Shetland sheepdog	0.98 (0.67, 1.44)	
Australian shephard	0.69 (0.46, 1.04)	
Other	Reference	
**Growth plate x-rays**		0.004
Not done	Reference	
Done at least once	1.38 (1.11, 1.73)	
**Handler current age**		0.003
18–24	Reference	
25–34	0.93 (0.59, 1.48)	
35–44	1.10 (0.69, 1.73)	
45–54	1.01 (0.65, 1.58)	
55–64	0.74 (0.47, 1.14)	
65+	0.56 (0.34, 0.90)	
**Handler medical training/experience**		0.012
None of these	Reference	
Veterinarian	0.69 (0.40, 1.17)	
Licensed vet tech	0.93 (0.53, 1.61)	
Veterinary assistant	2.10 (1.30, 3.42)	
Human health care professional	1.18 (0.92, 1.52)	

## Discussion

Several variables associated with either increased or decreased odds of injury in agility dogs were identified in this study. Some risk factors that were identified in previous North American surveys were also consistent in our North American survey sample. However, patterns were not entirely consistent between geographic regions.

The most consistent finding is that the Border Collie breed had the highest odds of injury, in both North American and non-North American samples when considering only severe injuries. This result is consistent with previous studies (5, 6). Border Collies are known for their fast speed; speed has been associated with increased odds of injury in equine athletes and in racing greyhounds, though no studies have been performed to assess if this is true in canines overall or in a particular breed or sport (7–9). Additional studies focused on Border Collies and correlation between structure, speed and injury, as well as severity of injury, are needed for further assessment given the robustness of this association seen in our survey and the number of Border Collies competing in agility worldwide.

Handler age above 65 was associated with lower risk of injury in the overall sample, and both the North American and non-North American samples, although it has not been noted in previous studies. Additionally, handlers over the age of 65 had the lowest risk of reporting a severe injury. There was a somewhat elevated risk for middle aged (25–44) aged handlers relative to the youngest category (18–24). It is possible that younger handlers are choosing faster dogs, and training at a higher intensity in order to keep up with the increased competitiveness and faster course times that have arisen over the last decade. Further studies are needed to assess training and handling styles of different age groups and how that might be associated with injury development and severity of injury.

Handler medical experience was correlated with injury risk, though correlation varied between geographic regions. Dogs with handlers that had medical training, especially those that were veterinarians or veterinary technicians, had a decreased risk of injury, with the exception of veterinary assistants. This held true in the overall sample as well as the non-North American sample, but was not true for handlers with medical experience in the North American sample. Veterinarians in particular, were also at the lowest risk of reporting a severe injury to their dogs. Those with a more advanced veterinary background may be able to detect subtle lameness or changes to gait more easily, and take appropriate measures to prevent further injury, thereby also decreasing the risk of severe injury. They may also be more aware of injury risk in general and take additional proactive approaches to injury prevention. It is unknown why there was a difference in risk between the non-North American and North American samples.

The North American sample, in this study, showed the same previous association with handler experience where dogs of handlers with greater agility experience were found to have a decreased risk for injury (1). However, the non-North American sample did not show this pattern at all, even in unadjusted models. Handler experience was also not associated with risk of severe injury. Inherently, having more experience as a handler should facilitate better handling techniques and timing of cues, which may improve safety in course navigation and decrease injury rates. In the equine literature it has been demonstrated that increased jockey experience is associated with decreased horse falls during steeplechase and point-to-point racing (10, 11). Proposed causes for this finding in horse racing include increased jockey skill in navigating the horse through the courses and quality of jockey training (10, 11). More experience as a handler may also lead to quicker identification in subtle changes in the athlete, allowing them to adjust training or seek veterinary care prior to significant injury. However, it is unknown why this correlation between increased experience and decreased injury risk did not hold true in the non-North American sample, even though the distribution of handler experience was similar between geographic region samples. It is possible that selection bias was more pronounced in the non-North American sample, resulting in only the more serious competitors filling out the survey, regardless of experience level. It is also possible that there are other confounding variables that either make experience level in the North American sample look protective, or non-protective in the non-North American sample. Prospective studies are needed to further elucidate the correlation between handler agility experience and injury risk, as well as how experience influences training, handling and competition factors that could also be involved with injury development and risk.

Dogs of handlers who had a history of competing at a national level had an increased risk of injury. This risk factor was present in the overall sample and North American sample, but not in the non-North American sample. There was no association found between competing at a national level and severity of injury. It is likely that handlers with a history of competing at the national level are selecting dogs for agility specifically, which was also an identified injury risk factor. They may also be training more frequently, for more repetitions and pushing their dogs harder during training and competitions, which could potentially increase injury risk. Overuse and repetitive stress injuries are common among human athletes and often related to high training frequency, intensity, and repetitive movements (12–14). With a retrospective survey we were unable to evaluate many of the training subtleties that could influence injury development. Prospective studies looking at specific training practices of dogs competing at the national level are needed to evaluate risks of overuse and repetitive stress injuries, as are often seen in highly competitive human athletes (12–14). The difference between geographic regions could be due to the smaller sample size in the non-North American sample, or it is also possible that differences between geographic regions are due to varying terminology and definitions of national level competitions.

This study also found that, in the overall sample and North American sample, dogs acquired after 12 months of age were found to have decreased odds of injury. In the overall sample, those dogs for which agility was not the main focus also had a decreased risk of injury. Handlers and their dogs who are competing recreationally in agility likely do not train as hard, nor as often as those competing at a national level. It is also possible that, like in human athletics, early sport specialization increases the risk of injury, particularly risk of overuse injuries (15–17).

In the North American sample, for dogs of the same height, the odds of injury were higher for dogs that were heavier. This correlation was only identified in the North American sample and not in the non-North American sample. There was also no association with severity of injury. Unfortunately, given the nature of using a survey we were unable to accurately assess body condition score of dogs, and therefore cannot make conclusions regarding relation of body condition score and injury risk. Without accurate body condition scoring, it is impossible to determine whether the heavier dogs had an increased body condition score and were obese, or whether the increased weight was due to increased muscle mass. It is possible that those dogs that are heavier, in relation to their height, are less physically fit, and therefore are more likely to sustain an injury. This correlation has been described in human sports medicine (18), and may be true of canines as well. Increased weight, regardless of the fitness level, places increased stress on an athlete's joints, which could increase injury risk, even in fit animals, as is described in the human literature (19, 20). It is unknown why there was a difference between dog weight and injury risk in the North American and non-North American samples, however smaller sample size in the non-North American sample is likely a limitation. It is also possible that the variation between geographic regions could be due to different breed distribution. While these data were adjusted for breed, there may be differences in breeds making up the “mixed breed” and “other purebred” categories that could affect the results. Additional studies are needed to evaluate the effect of body condition score and physical fitness on injury development and risk in canine athletes.

An interesting finding in the overall and North American samples was a correlation between having had radiographs taken to confirm growth plate closure and increased odds of injury risk. These dogs were also more prone to a severe injury, keeping them out of competition or training for an extended period of time. The correlation between having radiographs taken to confirm growth plate closure and injury risk was not retained in the final model in the non-North American sample. Radiographs for assessment of growth plate closure were more commonly performed in the North American sample vs. the non-North American sample (22% of dogs vs. 11% of dogs, respectively). Therefore, it is possible that the correlation with injury risk is similar in both geographic regions, but that the smaller sample size in the non-North American sample limited the statistical significance.

It is thought that high impact training before growth plate closure may cause injury to the growth plates or contribute to developmental musculoskeletal disorders (21). In humans, it has been demonstrated that adolescent athletes are prone to physeal injury due to overuse, particularly during times of rapid growth (22). Recommendations for preventing physeal injury in human adolescents include limiting time spent on a particular sport, as well as 2–3 months without training or competition per calendar year (22). In the equine literature, a single paper from 1973, compared soundness between 2 year old racehorses with open vs. closed growth plates after a season of racing (23). The data suggested that racing with open growth plates did not result in an increase of unsoundness (23). However, no further growth plate-specific research has been performed in horses. In contrast to the human literature, research in racehorses has shown that horses racing or starting race training at older than 2 years had a higher risk of catastrophic musculoskeletal injury, possibly due to decreased ability to adapt to the dynamic strains placed on bone (24, 25). However, this may not be directly comparable to dogs, as the majority of catastrophic musculoskeletal injuries in horses are fractures (24). This is opposite of dogs competing in agility, where the most common injuries are soft tissue injuries (2).

There are anecdotal guidelines among agility trainers and competitors on general ages to start various training techniques, jump heights, and obstacle training. Growth plate closure is often used in the agility community to determine when to progress the intensity of training, increase the height of jumps, and start weave training, although there are no studies available to support the use of growth plate closure as a guideline in determining training progression. It is possible that the population of agility handlers that choose to have radiographs of their dogs made to assess growth plate closure do so in order to train harder, and at a younger age, which may be contributing to the increased injury risk. It is also possible that radiographic growth plate closure is not a good indicator of safety for increasing training intensity as it does not necessarily correlate with development and strength of the surrounding soft tissue structures, such as ligaments, tendons, and muscles, nor cartilage development (25, 26). Studies focusing on sports readiness in adolescents have found that most children are ready for participation in sports by the age of 12 (21). These studies not only consider physical aspects, but also include cognitive and psychosocial development as well, making it difficult to make any direct comparisons to our canine agility athletes. Within the equine literature, sport readiness is debated and the optimal level of exercise in young horses is unknown (25–27).

One can also consider that those handlers who had radiographs taken to assess growth plate closure may also represent a population that is more aware of potential injuries, and/or have more access to veterinary practitioners with sports medicine expertise, which may make them more likely to diagnose an injury. However, this would not explain why these dogs were also at a higher risk of severe injury, as one would think that earlier recognition would lead to a less severe injury and/or more rapid return to training and competition. Further studies specifically focusing on growth plate closure, canine sports readiness, effect of training intensity on musculoskeletal development and injuries are warranted.

One surprising finding was that, in the non-North American sample, dogs owned by handlers who were professional trainers had a significantly increased risk of injury. This association was not observed in the North American sample. It could be hypothesized that handlers who are professional trainers are more likely to push their dogs harder in both training and competition in order to achieve success at high levels of competition, as this could affect their reputation and client demand. However, if this hypothesis were generally true, it would be expected that the association with increased injury risk would be found in the overall sample, as well as the different geographic samples, which was not the case. It is unknown why dogs of North American professional trainers did not have an increased injury risk, but those of non-North American trainers did. It is likely that there are training and competition differences between the North American and non-North American professional trainers that could result in injury risk differences. Prospective studies are needed to evaluate differences in training and competing among professional agility trainers between different geographic regions, and how that may influence development of injury.

Counter to our hypothesis, early (<12 months of age) spay/neuter was not associated with injury risk in the North American, non-North American or combined samples. Early spay and neuter practices have been associated with increased risk of joint disorders in certain breeds (28). One previous study on agility demographics did demonstrate a correlation between early spay/neuter and increased risk of injury in agility dogs, though there were very small cell sizes for some groups (3). In the current survey, however, no association was found between spay/neuter status and injury risk, in any geographic region, despite larger sample sizes. Additional studies are needed for further evaluation of association between spay/neuter status and injury risk in the sport dog population specifically.

In a study by Sellon et al. removal of dewclaws was associated with increased risk of digit injury (29). As such, we hypothesized that dewclaw removal would be associated with increased injury risk in our study population. Conversely, in this survey, presence or absence of dewclaws was not found to be associated with injury risk. However, these data evaluated variables in relation to overall injury risk, as opposed to individual anatomic locations or types of injury. It is possible that dewclaw removal is associated with increased risk of injury of specific anatomic locations or types of injury, but not overall injury risk.

Kinematic studies in dogs have demonstrated the importance of tail movement in maintaining balance during treadmill locomotion (30). This may be even more pronounced during advanced movements, such as performance of agility obstacles. We hypothesized that the resultant biomechanical changes from the absence of a tail may increase forces on the dog's body, resulting in increased injury. However, in this survey, presence or absence of tail was not found to be associated with injury risk. Biomechanical studies are needed to evaluate differences in movement patterns in relation to presence/absence tails, and how these movement patterns are associated with injury development and risk.

Limitations of this study include potential recall bias due to retrospectively collected data *via* survey. These data were also reported by handlers and were unable to be verified by a veterinarian, which could result in inaccuracies in injury data. Selection bias may also be present, so our findings may not be representative of the entire agility dog population. Data on severity of injury was based on time off from agility, which may not be an accurate depiction of the true severity of an injury due to other extenuating variables. Many of the observed associations in the North American sample that did not persist in the non-North American sample are likely sample size issues (given that there were similar trends seen outside of North America in the unadjusted models, but the other countries had smaller sample size).

Overall, this study confirmed previously reported increased risk for injury for Border Collies, and decreased risk for injury with greater handler experience. This study also identified new variables affecting risk for injury such as dog weight in relation to height, age at which the dog was acquired, agility as main sport focus, radiographic assessment for growth plate closure, handler medical training, handler occupation, and handler history of competition at a national level. Furthermore, this study identified differences in demographic risk factors between the North American population of handlers and the non-North American population of handlers. The results of the current study provide insight on risk factors for injury, as well as a basis to guide further research. More research is needed to evaluate the increased injury risk in Border Collies, how weight and body condition affect injury risk in canine athletes, how canine musculoskeletal development is impacted by training, and how handling factors impact injury risk. Knowledge of what risk factors exist for injury in agility dogs will aid in creating recommendations for training and veterinary care in order to help decrease injury in this population of dogs.

## Data Availability Statement

The raw data supporting the conclusions of this article will be made available by the authors, without undue reservation.

## Ethics Statement

Ethical review and approval was not required for the animal study because the Ohio State University Office of Responsible Research Practices determined the project was exempt from IRB review because it was an owner-based internet survey and the information was recorded without direct or indirect identifiers. The patients/participants provided their written informed consent to participate in this study.

## Author Contributions

ASu participated in data evaluation and writing the manuscript. AP and NK assisted in study design, data collection, data evaluation, and writing the manuscript. ASh participated in study design, statistical analysis, and writing the manuscript. All authors contributed to the article and approved the submitted version.

## Conflict of Interest

The authors declare that the research was conducted in the absence of any commercial or financial relationships that could be construed as a potential conflict of interest.

## Publisher's Note

All claims expressed in this article are solely those of the authors and do not necessarily represent those of their affiliated organizations, or those of the publisher, the editors and the reviewers. Any product that may be evaluated in this article, or claim that may be made by its manufacturer, is not guaranteed or endorsed by the publisher.

## Footnotes

^1^Personal communication. Carrie DeYoung, Director of AKC Agility. Email. June 30, 2020.
