# Supplementary material for: Internet Survey Evaluation of Demographic Risk Factors for Injury in Canine Agility Athletes
Source: Front Vet Sci. 2022 Apr 8;9:869702. doi: 10.3389/fvets.2022.869702 (PMC9024059; doi:10.3389/fvets.2022.869702)
Supplement: Supplementary file 1 [file Table_1.docx]

Supplemental Table 1. Age adjusted relationships between demographic variables and injury history in the full sample.

|  | Any injury  OR (95% CI) | Any injury  p-value | Severe injury  OR (95% CI) | Severe injury  p-value |
| --- | --- | --- | --- | --- |
| **Dog demographics** |  |  |  |  |
| Height & Weight together |  | 0.010 ^a^ |  | 0.26 |
| Dog Height (per 4 inches taller) | 1.05 (0.94, 1.18) |  | 1.08 (0.92, 1.27) |  |
| Dog Weight (per 10 pounds heavier) | 1.03 (0.96, 1.10) |  | 0.99 (0.90, 1.09) |  |
| Breed |  | <0.001^a^ |  | <0.001^a^ |
| Border Collie | 1.91 (1.63, 2.24) |  | 1.74 (1.42, 2.14) |  |
| Mixed Breed | 1.02 (0.83, 1.24) |  | 0.84 (0.63, 1.12) |  |
| Shetland Sheepdog | 1.05 (0.81, 1.37) |  | 0.95 (0.65, 1.38) |  |
| Australian Shephard | 1.01 (0.77, 1.31) |  | 0.70 (0.47, 1.06) |  |
| Other | REFERENCE |  | REFERENCE |  |
| Country/Region |  | <0.001^a^ |  | 0.49 |
| United States | REFERENCE |  | REFERENCE |  |
| Canada | 1.04 (0.83, 1.30) |  | 0.85 (0.62, 1.16) |  |
| UK / Ireland | 1.32 (1.07, 1.61) |  | 1.08 (0.81, 1.43) |  |
| Cont. Europe | 1.34 (1.06, 1.69) |  | 0.85 (0.60, 1.21) |  |
| Australia | 1.76 (1.27, 2.43) |  | 1.21 (0.79, 1.86) |  |
| Other | 1.47 (1.13, 1.91) |  | 1.20 (0.84, 1.72) |  |
| Age brought dog home |  | 0.007^a^ |  | 0.21 |
| <8 weeks | 0.98 (0.84, 1.16) |  | 0.90 (0.72, 1.13) |  |
| 8-12 weeks | REFERENCE |  | REFERENCE |  |
| 13-15 weeks | 0.99 (0.75, 1.31) |  | 1.05 (0.72, 1.53) |  |
| 4-6 months | 0.87 (0.66, 1.15) |  | 1.05 (0.73, 1.52) |  |
| 7-12 months | 0.91 (0.69, 1.20) |  | 0.95 (0.65, 1.38) |  |
| >12 months | 0.62 (0.49, 0.79) |  | 0.65 (0.46, 0.91) |  |
| How acquired |  | 0.059^a^ |  | 0.39 |
| Breeder | REFERENCE |  | REFERENCE |  |
| Rescue / Shelter | 0.82 (0.69, 0.97) |  | 0.85 (0.62, 1.15) |  |
| Other | 1.01 (0.81, 1.26) |  | 0.88 (0.70, 1.12) |  |
| Acquired w/agility in mind |  | 0.08^a^ |  | 0.09^a^ |
| No | 0.88 (0.77, 1.02) |  | 0.85 (0.70, 1.03) |  |
| Yes | REFERENCE |  | REFERENCE |  |
| Agility main sport focus |  | 0.016^a^ |  | 0.22 |
| Yes | REFERENCE |  | 0.99 (0.79, 1.24) |  |
| Mostly | 1.03 (0.88, 1.21) |  | 1.29 (0.96, 1.74) |  |
| No | 0.72 (0.57, 0.91) |  | REFERENCE |  |
| Sex / Neuter status |  | 0.50 |  | 0.70 |
| Male, Intact | REFERENCE |  | REFERENCE |  |
| Female, Intact | 0.88 (0.69, 1.13) |  | 1.02 (0.71, 1.48) |  |
| Male, Neutered <10 months | 1.07 (0.82, 1.39) |  | 1.16 (0.81, 1.66) |  |
| Male, Neutered 10-18 months | 0.95 (0.75, 1.20) |  | 0.88 (0.62, 1.24) |  |
| Male, Neutered >24 months | 0.92 (0.71, 1.19) |  | 0.96 (0.67, 1.37) |  |
| Female, Spayed <1 cycle | 1.17 (0.92, 1.49) |  | 1.20 (0.87, 1.66) |  |
| Female, Spayed 1 cycle | 0.97 (0.74, 1.27) |  | 0.95 (0.65, 1.39) |  |
| Female, Spayed >1 cycle | 1.02 (0.81, 1.29) |  | 1.04 (0.75, 1.44) |  |
| Front dew claws |  | 0.008^a^ |  | 0.016^a^ |
| Intact | REFERENCE |  | REFERENCE |  |
| Removed | 0.83 (0.72, 0.95) |  | 0.85 (0.70, 1.03) |  |
| Unknown | 0.61 (0.36, 1.03) |  | 0.28 (0.10, 0.78) |  |
| Rear dew claws |  | 0.69 |  | 0.56 |
| Intact | 0.95 (0.81, 1.12) |  | 0.88 (0.70, 1.11) |  |
| Removed or born without | REFERENCE |  | REFERENCE |  |
| Unknown | 0.89 (0.64, 1.24) |  | 0.92 (0.59, 1.45) |  |
| Docked tail |  | 0.001^a^ |  | 0.007^a^ |
| Yes | 0.74 (0.63, 0.88) |  | 0.72 (0.57, 0.92) |  |
| No / unknown | REFERENCE |  | REFERENCE |  |
| Growth plate x-rays |  | 0.007^a^ |  | 0.001^a^ |
| Not done | REFERENCE |  | REFERENCE |  |
| Done at least once | 1.25 (1.06, 1.47) |  | 1.43 (1.16, 1.77) |  |
| **Handler demographics** |  |  |  |  |
| Handler current age |  | <0.001^a^ |  | 0.001^a^ |
| 18-24 | REFERENCE |  | REFERENCE |  |
| 25-34 | 1.15 (0.83, 1.59) |  | 0.86 (0.54, 1.34) |  |
| 35-44 | 1.19 (0.86, 1.66) |  | 1.03 (0.66, 1.60) |  |
| 45-54 | 1.02 (0.75, 1.41) |  | 0.97 (0.63, 1.49) |  |
| 55-64 | 0.87 (0.64, 1.19) |  | 0.70 (0.46, 1.07) |  |
| 65+ | 0.57 (0.40, 0.79) |  | 0.55 (0.35, 0.88) |  |
| Handler gender |  | 0.43 |  | 0.64 |
| Female | REFERENCE |  | REFERENCE |  |
| Male | 0.85 (0.64, 1.14) |  | 0.82 (0.54, 1.23) |  |
| Other gender identity | 0.80 (0.43, 1.47) |  | 0.99 (0.44, 2.26) |  |
| Handler education |  | 0.51 |  | 0.043^a^ |
| Graduate or professional degree | REFERENCE |  | REFERENCE |  |
| 4-year college | 1.10 (0.94, 1.29) |  | 1.07 (0.86, 1.33) |  |
| 2-year college | 1.20 (0.96, 1.49) |  | 1.42 (1.07, 1.88) |  |
| Some college | 1.04 (0.85, 1.27) |  | 0.83 (0.62, 1.11) |  |
| HS degree (or less) | 1.10 (0.88, 1.38) |  | 1.02 (0.74, 1.41) |  |
| Handler profession |  | <0.001^a^ |  | 0.009^a^ |
| Not a dog trainer | REFERENCE |  | REFERENCE |  |
| Paid trainer, not primary job | 1.36 (1.17, 1.57) |  | 1.25 (1.03, 1.53) |  |
| Professional trainer | 1.32 (1.05, 1.66) |  | 1.46 (1.09, 1.97) |  |
| Handler medical training / experience |  | 0.062^a^ |  | 0.003^a^ |
| None of these | REFERENCE |  | REFERENCE |  |
| Veterinarian | 0.64 (0.45, 0.92) |  | 0.81 (0.48, 1.37) |  |
| Licensed vet tech | 0.83 (0.55, 1.25) |  | 1.12 (0.66, 1.93) |  |
| Veterinary assistant | 1.34 (0.89, 2.02) |  | 2.45 (1.53, 3.93) |  |
| Human health care professional | 0.94 (0.78, 1.13) |  | 1.20 (0.94, 1.55) |  |
| Handler agility experience |  | 0.018^a^ |  | 0.066^a^ |
| <3 years | 0.96 (0.75, 1.23) |  | 0.56 (0.36, 0.87) |  |
| 3-5 years | 1.14 (0.94, 1.38) |  | 1.09 (0.84, 1.42) |  |
| 6-10 years | 1.27 (1.07, 1.50) |  | 0.95 (0.76, 1.19) |  |
| 11-15 years | 1.26 (1.04, 1.53) |  | 1.05 (0.82, 1.35) |  |
| >15 years | REFERENCE |  | REFERENCE |  |
| Handler competed at national level |  | <0.001^a^ |  | 0.060^a^ |
| No | REFERENCE |  | REFERENCE |  |
| Yes | 1.27 (1.12, 1.44) |  | 1.19 (0.99, 1.42) |  |
| Handler competed at international level |  | <0.001^a^ |  | 0.55 |
| No | REFERENCE |  | REFERENCE |  |
| Yes | 1.47 (1.20, 1.80) |  | 1.09 (0.83, 1.44) |  |

^a^p<0.20 and included in initial adjusted model

Supplemental Table 2. Age adjusted relationships between demographic variables and injury history in the North American (NA) and non-North American (non-NA) samples.

|  | North American  OR (95% CI) | NA  p-value | Non-NA  OR (95% CI) | Non-NA  p-value |
| --- | --- | --- | --- | --- |
| **Dog demographics** |  |  |  |  |
| Height & Weight together |  | 0.028^a^ |  | 0.19^a^ |
| Dog Height (per 4 inches taller) | 1.04 (0.90, 1.20) |  | 1.02 (0.84, 1.23) |  |
| Dog Weight (per 10 pounds heavier) | 1.04 (0.95, 1.13) |  | 1.07 (0.94, 1.21) |  |
| Breed |  | <0.001^a^ |  | <0.001^a^ |
| Border Collie | 1.70 (1.39, 2.07) |  | 2.23 (1.69, 2.93) |  |
| Mixed Breed | 0.85 (0.67, 1.08) |  | 1.46 (1.03, 2.07) |  |
| Shetland Sheepdog | 1.07 (0.79, 1.45) |  | 1.01 (0.61, 1.70) |  |
| Australian Shephard | 0.95 (0.71, 1.27) |  | 1.47 (0.77, 2.81) |  |
| Other | REFERENCE |  | REFERENCE |  |
| Country/Region |  | 0.74 |  | 0.45 |
| United States | REFERENCE |  |  |  |
| Canada | 1.02 (0.91, 1.14) |  |  |  |
| UK / Ireland |  |  | REFERENCE |  |
| Cont. Europe |  |  | 1.02 (0.76, 1.35) |  |
| Australia |  |  | 1.33 (0.92, 1.92) |  |
| Other |  |  | 1.12 (0.82, 1.53) |  |
| Age brought dog home |  | 0.004^a^ |  | 0.55 |
| <8 weeks | 0.95 (0.78, 1.16) |  | 1.06 (0.79, 1.41) |  |
| 8-12 weeks | REFERENCE |  | REFERENCE |  |
| 13-15 weeks | 1.14 (0.81, 1.58) |  | 0.72 (0.42, 1.22) |  |
| 4-6 months | 0.91 (0.65, 1.27) |  | 0.79 (0.48, 1.32) |  |
| 7-12 months | 1.01 (0.74, 1.39) |  | 0.69 (0.38, 1.25) |  |
| >12 months | 0.57 (0.43, 0.75) |  | 0.93 (0.57, 1.51) |  |
| How acquired |  | 0.077^a^ |  | 0.78 |
| Breeder | REFERENCE |  | REFERENCE |  |
| Rescue / Shelter | 1.04 (0.79, 1.36) |  | 0.93 (0.64, 1.35) |  |
| Other | 0.80 (0.65, 0.98) |  | 0.90 (0.65, 1.25) |  |
| Acquired w/agility in mind |  | 0.034 ^a^ |  | 0.44 |
| No | 0.83 (0.70, 0.99) |  | 0.91 (0.71, 1.16) |  |
| Yes | REFERENCE |  | REFERENCE |  |
| Agility main sport focus |  | 0.035^a^ |  | 0.58 |
| Yes | REFERENCE |  | REFERENCE |  |
| Mostly | 1.03 (0.85, 1.24) |  | 1.09 (0.81, 1.48) |  |
| No | 0.71 (0.54, 0.93) |  | 0.83 (0.52, 1.33) |  |
| Sex / Neuter status |  | 0.30 |  | 0.61 |
| Male, Intact | REFERENCE |  | REFERENCE |  |
| Female, Intact | 0.77 (0.56, 1.07) |  | 1.03 (0.69, 1.53) |  |
| Male, Neutered <10 months | 1.08 (0.79, 1.48) |  | 1.09 (0.66, 1.80) |  |
| Male, Neutered 10-18 months | 0.95 (0.71, 1.26) |  | 1.00 (0.65, 1.54) |  |
| Male, Neutered >24 months | 0.83 (0.61, 1.14) |  | 1.25 (0.78, 1.99) |  |
| Female, Spayed <1 cycle | 1.11 (0.84, 1.48) |  | 1.49 (0.95, 2.32) |  |
| Female, Spayed 1 cycle | 0.91 (0.65, 1.27) |  | 1.05 (0.67, 1.64) |  |
| Female, Spayed >1 cycle | 1.08 (0.81, 1.43) |  | 0.90 (0.59, 1.38) |  |
| Front dew claws |  | 0.33 |  | 0.063^a^ |
| Intact | REFERENCE |  | REFERENCE |  |
| Removed | 0.95 (0.81, 1.11) |  | 0.62 (0.39, 0.97) |  |
| Unknown | 0.63 (0.33, 1.20) |  | 0.60 (0.25, 1.43) |  |
| Rear dew claws |  | 0.74 |  | 0.096^a^ |
| Intact | 0.95 (0.75, 1.19) |  | 0.76 (0.59, 0.98) |  |
| Removed or born without | REFERENCE |  | REFERENCE |  |
| Unknown | 0.87 (0.59, 1.31) |  | 0.83 (0.47, 1.47) |  |
| Docked tail |  | 0.054^a^ |  | 0.035^a^ |
| Yes | 0.84 (0.70, 1.00) |  | 0.55 (0.31, 0.96) |  |
| No / unknown | REFERENCE |  | REFERENCE |  |
| Growth plate x-rays |  | 0.003^a^ |  | 0.15^a^ |
| Not done | REFERENCE |  | REFERENCE |  |
| Done at least once | 1.32 (1.10, 1.59) |  | 1.31 (0.91, 1.88) |  |
| **Handler demographics** |  |  |  |  |
| Handler current age |  | <0.001^a^ |  | 0.15^a^ |
| 18-24 | REFERENCE |  | REFERENCE |  |
| 25-34 | 1.09 (0.67, 1.77) |  | 1.29 (0.83, 2.01) |  |
| 35-44 | 1.28 (0.79, 2.07) |  | 1.17 (0.74, 1.84) |  |
| 45-54 | 1.18 (0.74, 1.87) |  | 0.91 (0.58, 1.44) |  |
| 55-64 | 0.95 (0.60, 1.49) |  | 1.01 (0.63, 1.62) |  |
| 65+ | 0.62 (0.38, 0.99) |  | 0.70 (0.39, 1.27) |  |
| Handler gender |  | 0.08^a^ |  | 0.81 |
| Female | REFERENCE |  | REFERENCE |  |
| Male | 0.68 (0.46, 0.99) |  | 1.10 (0.69, 1.76) |  |
| Other gender identity | 0.67 (0.32, 1.39) |  | 1.37 (0.41, 4.63) |  |
| Handler education |  | 0.50 |  | 0.54 |
| Graduate or professional degree | REFERENCE |  | REFERENCE |  |
| 4-year college | 1.11 (0.92, 1.33) |  | 1.16 (0.85, 1.59) |  |
| 2-year college | 1.15 (0.90, 1.48) |  | 1.48 (0.94, 2.32) |  |
| Some college | 0.99 (0.78, 1.26) |  | 1.12 (0.78, 1.61) |  |
| HS degree (or less) | 0.88 (0.63, 1.24) |  | 1.13 (0.82, 1.57) |  |
| Handler profession |  | 0.10 ^a^ |  | <0.001 ^a^ |
| Not a dog trainer | REFERENCE |  | REFERENCE |  |
| Paid trainer, not primary job | 1.18 (0.98, 1.41) |  | 1.61 (1.25, 2.06) |  |
| Professional trainer | 1.23 (0.95, 1.60) |  | 1.78 (1.09, 2.89) |  |
| Handler medical training / experience |  | 0.33 |  | 0.15^a^ |
| None of these | REFERENCE |  | REFERENCE |  |
| Veterinarian | 0.69 (0.45, 1.07) |  | 0.53 (0.28, 1.02) |  |
| Licensed vet tech | 0.97 (0.62, 1.51) |  | 0.50 (0.17, 1.49) |  |
| Veterinary assistant | 1.38 (0.84, 2.28) |  | 1.24 (0.59, 2.59) |  |
| Human health care professional | 1.03 (0.83, 1.28) |  | 0.80 (0.55, 1.16) |  |
| Handler agility experience |  | 0.003^a^ |  | 0.89 |
| <3 years | 0.87 (0.62, 1.21) |  | 0.86 (0.57, 1.29) |  |
| 3-5 years | 1.18 (0.93, 1.49) |  | 0.88 (0.62, 1.24) |  |
| 6-10 years | 1.38 (1.13, 1.68) |  | 0.91 (0.65, 1.26) |  |
| 11-15 years | 1.33 (1.06, 1.65) |  | 1.02 (0.69, 1.49) |  |
| >15 years | REFERENCE |  | REFERENCE |  |
| Handler competed at national level |  | 0.007^a^ |  | 0.022^a^ |
| No | REFERENCE |  | REFERENCE |  |
| Yes | 1.24 (1.06, 1.44) |  | 1.31 (1.04, 1.66) |  |
| Handler competed at international level |  | 0.21 |  | 0.008^a^ |
| No | REFERENCE |  | REFERENCE |  |
| Yes | 1.21 (0.90, 1.64) |  | 1.48 (1.11, 1.97) |  |

^a^p<0.20 and included in initial adjusted model
